# The Relationship of the Facial Nerve to the Condylar Process: A Cadaveric Study with Implications for Open Reduction Internal Fixation

**DOI:** 10.1155/2015/715126

**Published:** 2015-09-03

**Authors:** H. P. Barham, P. Collister, V. D. Eusterman, A. M. Terella

**Affiliations:** ^1^Department of Otolaryngology-Head and Neck Surgery, Louisiana State University Health Sciences Center, 533 Bolivar Street, Suite 566, New Orleans, LA 70112, USA; ^2^Department of Otolaryngology-Head and Neck Surgery, University of Colorado, Aurora, CO, USA

## Abstract

*Introduction*. The mandibular condyle is the most common site of mandibular fracture. Surgical treatment of condylar fractures by open reduction and internal fixation (ORIF) demands direct visualization of the fracture. This project aimed to investigate the anatomic relationship of the tragus to the facial nerve and condylar process. *Materials and Methods*. Twelve fresh hemicadavers heads were used. An extended retromandibular/preauricular approach was utilized, with the incision being based parallel to the posterior edge of the ramus. Measurements were obtained from the tragus to the facial nerve and condylar process. *Results*. The temporozygomatic division of the facial nerve was encountered during each approach, crossing the mandible at the condylar neck. The mean tissue depth separating the facial nerve from the condylar neck was 5.5 mm (range: 3.5 mm–7 mm, SD 1.2 mm). The upper division of the facial nerve crossed the posterior border of the condylar process on average 2.31 cm (SD 0.10 cm) anterior to the tragus. *Conclusions*. This study suggests that the temporozygomatic division of the facial nerve will be encountered in most approaches to the condylar process. As visualization of the relationship of the facial nerve to condyle is often limited, recognition that, on average, 5.5 mm of tissue separates condylar process from nerve should help reduce the incidence of facial nerve injury during this procedure.

## 1. Introduction

The condylar process has been reported as most common site of mandibular fractures, accounting for 29% of all mandibular fractures [1]. Surgical treatment of condylar fractures by open reduction and internal fixation (ORIF) demands that internal fixation and anatomic reduction be completed under direct visualization of the fracture. One challenge of open surgery for condylar process fractures is navigating the anatomic complexity of the adjacent vital structures, specifically the facial nerve.

Several authors have described the location of the facial nerve in the preauricular area. Despite this, one of the most common complications of open reduction and internal fixation of subcondylar fractures remains facial nerve paresis and paralysis [2–4].

It is our feeling that the novice surgeon can benefit from a system of reference to enable the prediction of critical anatomic structures. This system must be based on anatomical landmarks that are (1) easily identifiable, (2) fixed in position during the procedure, and (3) independent of patient position [2].

This project aimed to describe pertinent anatomic relationships of the facial nerve in the preauricular region and relate these findings to ORIF procedures of the subcondylar region. Specifically, we describe the anatomic relationship of the nerve to subcondylar mandible and to easily palpable topographic landmarks, such as the tragus. We feel that these relationships are especially germane to the less experienced surgeon performing ORIF for condylar process fractures.

## 2. Materials and Methods

Twelve hemicadavers heads were used. An extended preauricular/retromandibular approach was utilized to provide broad exposure of the facial nerve and subcondylar region. The incision was based parallel to the posterior edge of the ramus. Once parotid tissue was encountered, blunt dissection was carried out to the facial nerve branches.

Because of the ease of palpation and fixed location during ORIF of the subcondylar region, the posterior apex of the tragus and the lateral pole of the condyle were used as reference points for measurements.

**Figure 1:**
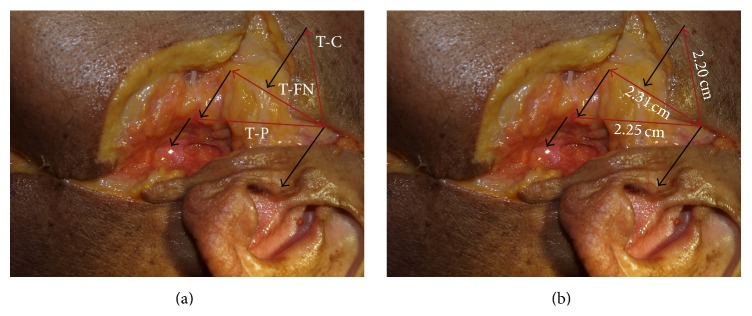
Illustrated in photo are the relationships of the temporozygomatic (upper) division of the facial nerve (FN) to the tragus (T), lateral pole of condyle (C), and pes anserinus (P).

Measurements were made as follows (Figure 1(a)):

1. Depth of tissue separating facial nerve from the underlying condylar neck.
2. Tragus (posterior apex) to the condyle (lateral pole).
3. Tragus (posterior apex) to the point where the facial nerve crossed the posterior border of the condylar neck.
4. Tragus (posterior apex) to the pes anserinus.

All dissections were performed by one of two authors (H. P. Barham or A. M. Terella). Measurements were made by one of the authors and verified independently by the other.

## 3. Results

The temporozygomatic (upper) division of the facial nerve was encountered during each of our dissections to the subcondylar region. This division of the facial nerve consistently emerged from posterior and medial to the condylar neck and traveled in an oblique plane. In all cases, this division crossed the mandible at the condylar neck. The mean depth from facial nerve to underlying condylar neck was 5.5 mm (standard deviation: 1.2 mm).

The mean distance from tragus (posterior apex) to condyle (lateral pole) was 2.20 cm (standard deviation: 0.04 cm), from tragus (posterior apex) to the point where the facial nerve crossed the posterior border of the condylar neck it was 2.31 cm (standard deviation: 0.10 cm), and from the tragus to pes anserinus it was 2.25 cm (standard deviation: 0.10 cm) (Figure 1(b)).

## 4. Discussion

An open approach to the treatment of condylar fractures has become increasingly common, and several surgical incisions including preauricular, rhytidectomy, retromandibular, submandibular, and postauricular incision have been described. A potential and devastating complication of ORIF in this region is facial paralysis or palsy. The reported incidence of facial nerve palsy varies widely, with a reported incidence of 0% utilizing a high submandibular approach to as high as 30% with a retromandibular approach [2–5] (Table 1). Our findings support those of authors prior in suggesting that the temporozygomatic division of the facial nerve has an intimate anatomic relationship to the condylar process. We attempt to expand on this work by highlighting the depth of tissue separating the nerve from the underlying condylar process. When approaching the condylar region from a retromandibular approach or preauricular approach, visualization of the facial nerve-condyle relationship is limited, and moderately strong retraction is frequently required to obtain an adequate visual field and working space for osteosynthesis. Although the temporozygomatic (upper) division of the facial nerve should not be encountered during the submandibular or high submandibular approaches, the nerve is retracted laterally and easily stretched when attempting to achieve an ample working space and optical field. On average, only 5.5 mm of tissue separates the condylar process from the nerve. The surgeon must appreciate that blind and aggressive lateral or superior retraction of overlying soft tissue in this region can easily result in stretch injury and neuropraxis. Understanding this close relationship should help reduce the incidence of facial nerve injury during ORIF of the condylar region.

Additionally, on average, the pes anserinus of the facial nerve was located approximately 2.25 cm anterior-inferior to the tragus, while the facial nerve crossed the posterior border of the mandible on average 2.31 cm anterior-inferior to the tragus. The measurements and relationship of the facial nerve from this study should allow for nerve position to be estimated using the tragus and palpated posterior border of the mandible.

It is our opinion that the use of a palpable landmark is of greatest utility to the novice surgeon, less experienced in this region. Techniques and measurements to predict nerve location are only estimates and cannot replace the need for precise anatomic understanding and cautious dissection in the condylar region. Further, they must be interpreted understanding the inherent, well documented anatomic variation of the facial nerve.

Several studies have demonstrated efficacy of techniques for locating the facial nerve, with the work of de Ru et al. being the most complete and showing the single best anatomic landmark for locating the facial nerve trunk to be the tympanomastoid fissure (TMF), usually within 3 mm of this landmark [6]. These findings were confirmed by Pather and Osman. However, Pather and Osman noted that the TMF was not an ideal landmark because it often lay behind the sturdy tendon of the sternocleidomastoid muscle, thus requiring a complex dissection [7]. These techniques are excellent in localizing the nerve during dissection but do not help provide a preoperative estimate of nerve location in the condylar region.

Limitations to this study include those that are common to any cadaveric anatomic study. Tissues operated upon following a traumatic insult may undergo distortion due to edema or disruption of soft tissues. Presumably, a swelling process would increase distances between structures if uniformly distributed, so it may not significantly change the surgeon's operative strategy. Further, it is acknowledged that even careful anatomic dissection could lead to distortion of tissue in our specimens, thus affecting measurements. Lastly, our limited sample size enabled calculation of standard deviations, but not an evaluation of anatomic variation.

## 5. Conclusions

The temporozygomatic (upper) division of the facial nerve has an intimate relationship to the condylar process. It is critical to understanding both the course of the nerve and the depth of tissue separating it from the condylar neck. Soft tissue retraction to optimize the optical field can easily stretch the nerve resulting in neuropraxis. Understanding this close relationship should help reduce the incidence of facial nerve injury during ORIF of the condylar region.

Further the novice surgeon, less experienced in the subcondylar region, can benefit from estimates of facial nerve location using easily palpable topographic landmarks. We suggest that the tragus and lateral pole of the condyle can serve this function.

## Figures and Tables

**Table 1:** Reported rate of facial nerve injury.

Researcher	Approach	Sample size	Rate of facial nerve injury
Pereira et al. [2]	Preauricular	21	30%
Hammer et al. [3]	Preauricular	31	3.2%
MacArthur et al. [4]	Preauricular	13	15.4%
Meyer et al. [5]	High submandibular	64	0%
